# Supplementary material for: Immunogenicity and Efficacy of TNX-1800, A Live Virus Recombinant Poxvirus Vaccine Candidate, against SARS-CoV-2 Challenge in Nonhuman Primates
Source: Vaccines (Basel). 2023 Nov 2;11(11):1682. doi: 10.3390/vaccines11111682 (PMC10674175; doi:10.3390/vaccines11111682)
Supplement: Supplementary file 1 [file vaccines-11-01682-s001.zip › vaccines-2666280-supplementary.pdf]

Supplementary Figures:

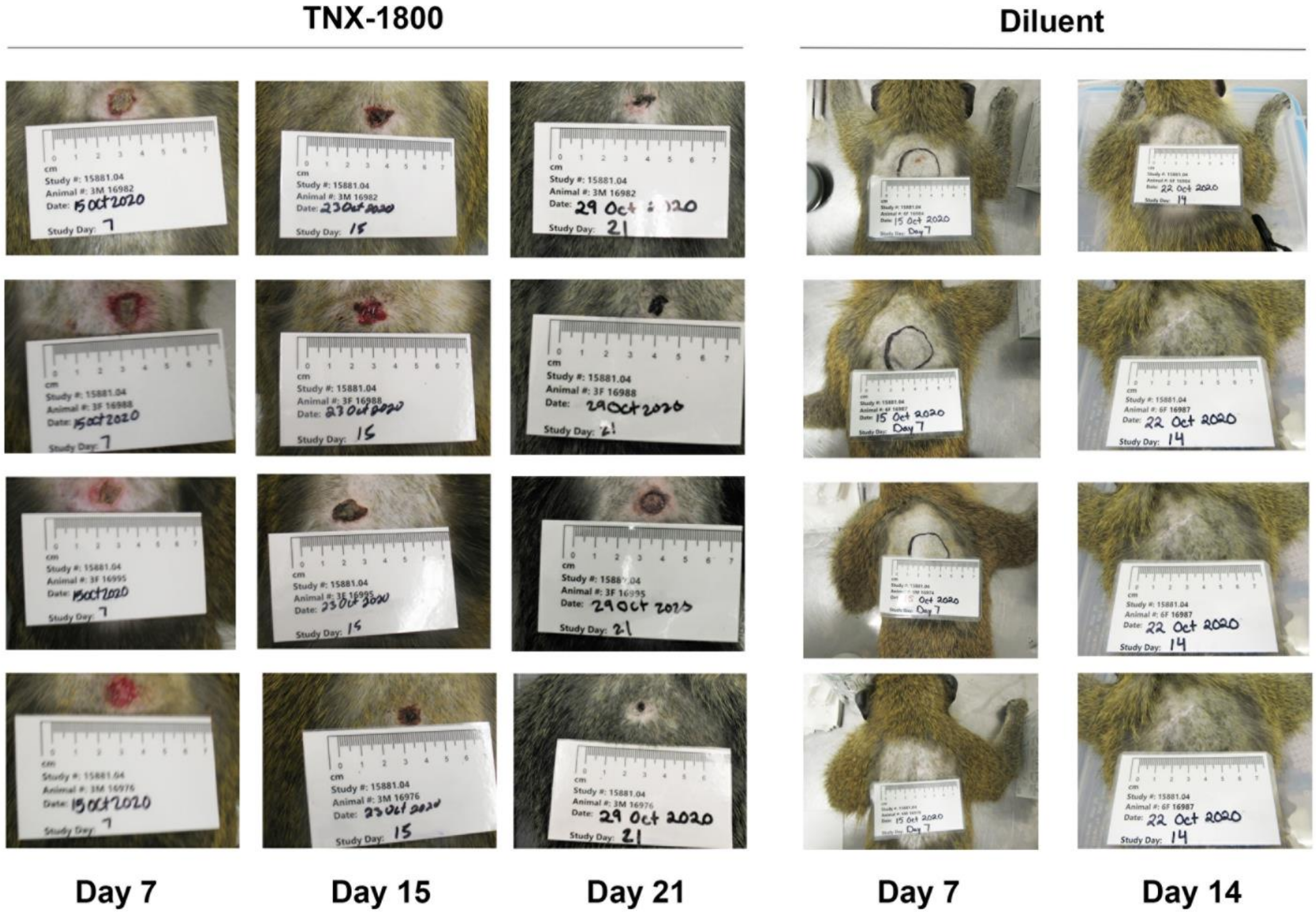

Figure S1. Picture of the vaccination site of the African Green Monkeys. Vaccination site lesions were assessed and photographed on Days 7, 14/15, and 21.

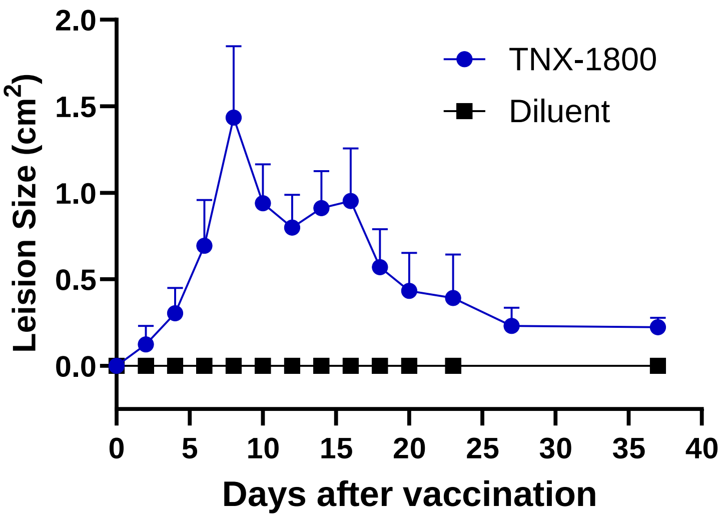

Figure S2. Lesion size measurements of the vaccination site of Cynomolgus Monkeys. Photographs were taken and measured to determine the lesion size. The entire area was measured including any remaining scar tissue. The data represents the mean lesion area (cm^2) from immunization (Study Day 0) until Study Day 37. The error bars for the group means represent the standard error of the mean (SEM).
